# Development and validation of LCMM prediction algorithms to estimate recovery pattern of postoperative AKI in type A aortic dissection: a retrospective study

**DOI:** 10.3389/fcvm.2024.1364332

**Published:** 2024-04-19

**Authors:** Weiwei Zhao, Ya-peng Wang, Xinlong Tang, Yi Jiang, Yunxing Xue, Yali Wang, Qiuju Ding, Huimei Chen, Dongjin Wang, YongQing Cheng, Min Ge, Qing Zhou

**Affiliations:** ^1^ Department of Cardiothoracic Surgery, Nanjing Drum Tower Hospital, Affiliated Hospital of Medical School, Nanjing University, Nanjing, Jiangsu, China; ^2^ Department of Cardiothoracic Surgery, Nanjing Drum Tower Hospital, Chinese Academy of Medical Sciences & Peking Union Medical College, Nanjing, Jiangsu, China; ^3^ Programme in Cardiovascular and Metabolic Disorders, Duke-NUS Medical School, Singapore, Singapore

**Keywords:** type A aortic dissection, acute kidney injury, recovery, trajectory, glomerular filtration rate

## Abstract

**Background:**

Postoperative acute kidney injury (PO-AKI) is a prevalent complication among patients with acute type A aortic dissection (aTAAD) for which unrecognized trajectories of renal function recovery, and their heterogeneity, may underpin poor success in identifying effective therapies.

**Methods:**

This was a retrospective, single-center cohort study in a regional Great Vessel Center including patients undergoing aortic dissection surgery. Estimated glomerular filtration rate (eGFR) recovery trajectories of PO-AKI were defined through the unsupervised latent class mixture modeling (LCMM), with an assessment of patient and procedural characteristics, complications, and early-term survival. Internal validation was performed by resampling.

**Results:**

A total of 1,295 aTAAD patients underwent surgery and 645 (49.8%) developed PO-AKI. Among the PO-AKI cohort, the LCMM identified two distinct eGFR trajectories: early recovery (ER-AKI, 51.8% of patients) and late or no recovery (LNR-AKI, 48.2% of patients). Binary logistic regression identified five critical determinants regarding poor renal recovery, including chronic kidney disease (CKD) history, renal hypoperfusion, circulation arrest time, intraoperative urine, and myoglobin. LNR-AKI was associated with increased mortality, continuous renal replacement therapies, mechanical ventilation, ICU stay, and hospital stay. The assessment of the predictive model was good, with an area under the curve (AUC) of 0.73 (95% CI: 0.69–0.76), sensitivity of 61.74%, and specificity of 75.15%. The internal validation derived a consistent average AUC of 0.73. The nomogram was constructed for clinicians' convenience.

**Conclusion:**

Our study explored the PO-AKI recovery patterns among surgical aTAAD patients and identified critical determinants that help to predict individuals at risk of poor recovery of renal function.

## Introduction

Acute type A aortic dissection (aTAAD) is a cardiovascular emergency characterized by high mortality and severe complications, even with timely surgical treatment (1, 2). Acute kidney injury (AKI) has been well realized as a common postoperative complication of aTAAD and is often associated with a poor prognosis (3–5), but advancement of effective interventions has been limited by unrecognized heterogeneity in recovery course and risk stratification. In fact, evident dissimilarity in the natural history of kidney injuries from multiple sources, including sepsis, type 2 diabetes, and kidney transplantation, has been observed (6, 7). Importantly, no study to date has been designed to delineate and analyze the longitudinal trajectories of PO-AKI in aTAAD patients.

Recently, unsupervised latent class mixture modeling (LCMM) has been introduced as an objective data-driven method for exploring homogeneous structures within heterogeneous data (8). This approach was successfully used to identify disease subtypes in disability, atherosclerosis, and end-stage renal disease (7, 9, 10), with an effect on clinical practice. In this study, we applied LCMM to develop and validate a prediction algorithm to estimate the PO-AKI recovery pattern in patients who underwent surgery for aTAAD. We used serial estimated glomerular filtration rate (eGFR) during the first 7 postoperative days to define recovery patterns and used the resultant variable to build a predictive model. The design of this study was to identify patients at different risk levels to provide a better diagnostic and prognostic approach and improve our ability to develop better strategies to deal with this complication.

## Methods

### Patient enrollment and study approvals

**Figure 1:**
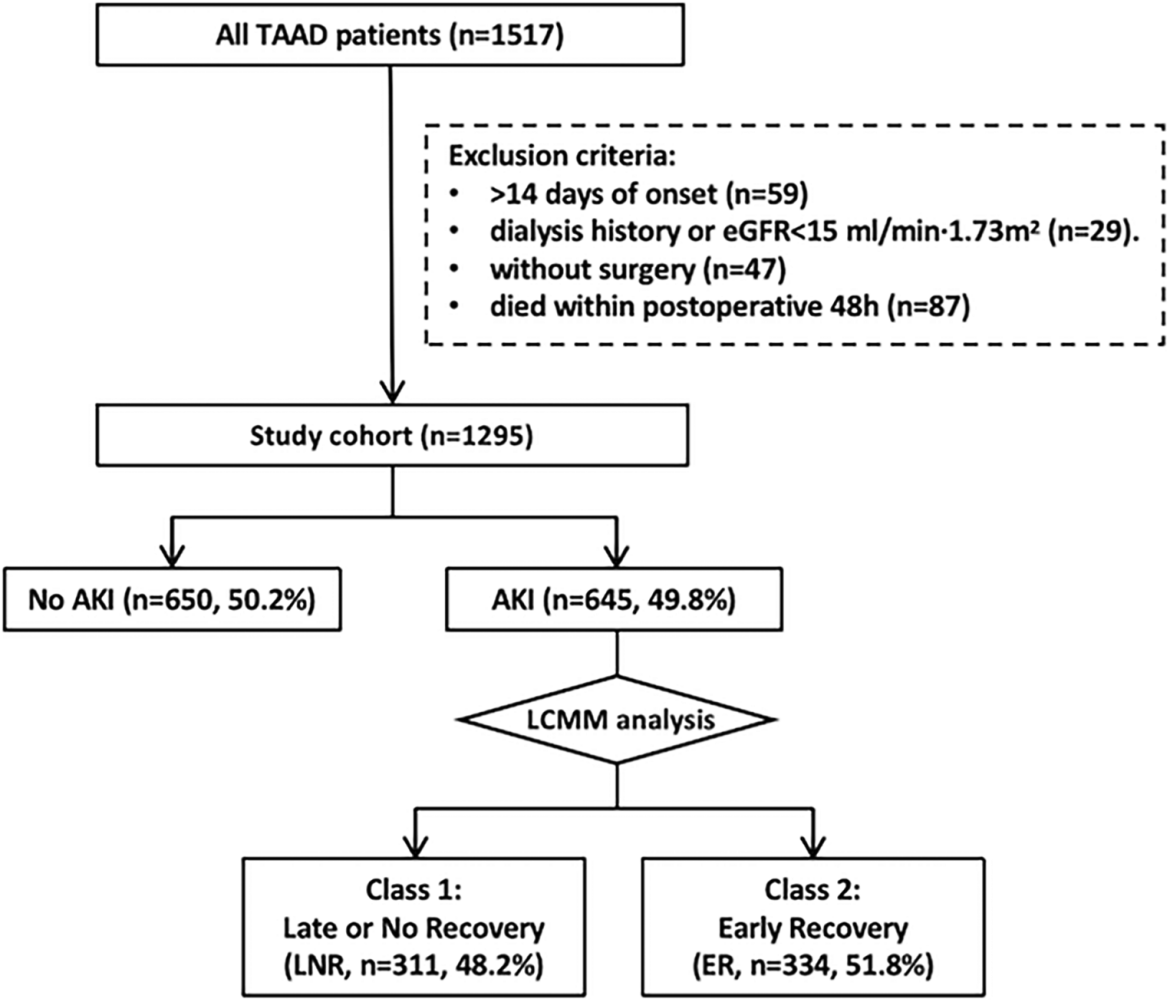
Workflow of patient selection and analysis. TAAD, type A aortic dissection; aTAAD, acute type A aortic dissection; eGFR, estimated glomerular filtration rate; AKI, acute kidney injury; LNR, late or no recovery; ER, early recovery; LCMM, latent class mixture model.

This study retrospectively screened 1,517 patients with CT-confirmed type A aortic dissection (TAAD), from the Department of Cardiac Surgery of Nanjing Drum Tower Hospital (China), Great Vessel Disease Diagnosis and Treatment Center of Jiangsu Province, between 2013 and 2021 (Figure 1). Among them, a total of 1,295 acute TAAD (aTAAD, ≤14 days of onset) patients who underwent open surgery were included in the study. The exclusion criteria were as follows: chronic TAAD (>14 days of onset, *n* = 59), without surgical treatment (refusal of surgery or preoperative rupture, *n* = 47), death occurred within postoperative 48 h (*n* = 87), prior chronic hemodialysis, peritoneal dialysis (lasting ≥3 months), or eGFR < 15 ml/min/1.73 m^2^ not receiving renal replacement therapy (*n* = 29).

Ethical approvals were set forth by the Ethics Committee of the Nanjing Drum Tower Hospital, in accordance with the Declaration of Helsinki and in respect of biosafety and public health. All methods and procedures were conducted according to the local instructions in line with the clinical guidelines. Written informed consent was obtained from every participant before clinical practice.

### Data collection and definition

The medical records and laboratory results of all patients were collected from the clinical database of the Nanjing Drum Tower Hospital. Preoperative data included demographical information, smoking and drinking history, complications [hypertension, cardiovascular disease and chronic kidney disease (CKD)], onset time, preoperative hypotension, organ malperfusion. Intraoperative data included surgery time, cardiopulmonary bypass (CPB) application and time, cross-clamp time, circulation arrest time, lowest temperature, cannulation strategy, stent implantation, and urine output. Postoperative data included myoglobin level (immediately after the procedures), duration of mechanical ventilation, and use of renal replacement treatment, and the short-term outcomes included intensive care unit (ICU) stay, hospitalization time, and 30-day mortality.

Postoperative acute kidney injury (PO-AKI) was assessed according to the Kidney Disease: Improving Global Outcomes (KDIGO) guidelines within postoperative 1 week, as any of the following: an increase in serum creatinine (SCr) by ≥0.3 mg/dl (≥26.5 μmol/L) within 48 h; an increase in SCr to ≥1.5-fold of the baseline value, which is known or presumed to have occurred within the prior 7 days; or urine volume <0.5 ml/kg/h for 6 h. Therein, patients were first grouped into the AKI group and the no-AKI group. Baseline renal function was defined as preoperative eGFR. The myoglobin limit of 700 ng/ml is approximately 10 times greater than the upper limit of normal (ULN) values for our clinical laboratory, a value that would eliminate from consideration a large number of patients with mild myoglobin level elevations and still provide a broad range of values to evaluate. Therefore, we defined the variable “myoglobin > 10 ULN” as serum myoglobin greater than 10 times the ULN (>700 ng/ml), tested immediately after the procedures.

### Imaging analysis

CT scanning imaging of all patients was carefully reviewed by at least two experienced radiologists and two cardiac surgeons with more than 10 years of diagnostic experience, who reached a consensus on the image analysis. Post-processing and measurement of CT perfusion images were conducted by the radiologists. The general assessment of CT imaging signs associated with dissection included different AD types, the origin of renal arteries, false lumen thrombosis, and the size, number, and position of entry tears. A comparison of renal perfusion imaging was performed by placing the region of interest (ROI) in the bilateral renal cortex and then measuring the mean CT values [Hounsfield unit (H)]. Five ROIs were assessed if the presence of hypo-enhanced areas was visually significant compared with the contralateral kidney. A difference of ≧20 in the mean Hounsfield units between the bilateral kidneys was defined as positive as conducted in a previously reported strategy (11).

### Data missingness

Variables with missing proportions exceeding 15% were deleted, and the missing variables and their proportions are presented in Supplementary Table S1. We utilized the “mice” software package based on multiple imputations, with the “random forest” method, to complete the imputation process. We generated five imputed datasets and selected the first one as the primary dataset for subsequent analyses. The distribution of the variables with missing values is illustrated in Supplementary Figure S1A, and the distribution of the original dataset and the imputed datasets are illustrated in Supplementary Figure S1B.

### Identification of PO-AKI recovery trajectories

We used the latent class mixture model (LCMM), implemented in the “lcmm” R package (R software, version 4.2.0, http://www.R-project.org) (8), which employs maximum likelihood parameter estimation to delineate the group-based longitudinal trajectories for repeated eGFR measures metrics. We explored the fit of LCMM with one to five trajectory classes (Supplementary Table S2), using both linear and natural spline function forms (three and four knots). The optimal trajectory class model was determined based on the following criteria: (1) using Akaike information criterion (AIC) and (2) Bayesian information criterion (BIC), where smaller values indicate better model fit; (3) ensuring that the number of samples assigned to each trajectory is no less than 10% of the total sample size; (4) requiring a mean posterior probability >70% for each trajectory class; and (5) considering the interpretability and research significance of the identified trajectories. To mitigate the risk of local maxima, an automatic grid search with 30 random initial values was utilized to fit the LCMM. The trajectory categories served as the outcome for subsequent binary regression analysis.

### Statistical analyses

Statistical analysis was performed using the IBM SPSS (version 25.0) and R software (version 4.2.0) (http://www.R-project.org). Normal distributed continuous variables were presented as means ± SD and compared by the *T*-test. Non-normal distributed continuous variables were presented as median [interquartile ranges (IQRs)] and compared by the Mann–Whitney *U*-test. Categorical variables were presented as counts (proportions) and compared by the chi-square test. Univariate and multivariate logistic regression analyses were applied to screen the independent predictors of trajectory subgroups, and the odds ratio (OR) and 95% confidence interval (95% CI) were also recorded. All variables in the univariate regression analysis were entered into the stepwise multivariate logistic regression analysis. A prediction model was established by adopting variables with statistical association and clinical significance and excluding variables with collinear correlation. Then, the performance of our prediction model was evaluated via internal validation in the original cohort by bootstrap (1,000 resamples). Diagnostic performance was evaluated by plotting receiver operating characteristic (ROC) curves and their values of area under the curve (AUC). The accuracy of the diagnostic test was evaluated by sensitivity, specificity, positive predictive values, and negative predictive values for different diagnostic criteria. *P* < 0.05 was considered statistically significant.

## Results

### Demographic characteristics of all patients

In total, 1,295 aTAAD patients who underwent open aortic surgery were incorporated in the retrospective study (Figure 1, Table 1). The mean ± SD age was 53.6 ± 13.3 years, and 75.4% (976/1,295) of the patients were male. The median (IQR) postoperative ICU stay and hospital stay were 5 (3, 8) days and 18 (14, 24) days, respectively, and the 30-day mortality rates were 7.0% (91/1,295). After surgery, 49.8% (645/1,295) of the patients developed postoperative AKI (PO-AKI). Compared with the no-AKI group, the duration of ICU stay and hospital stay of the AKI group were significantly prolonged (*P* < 0.001), and the 30-day mortality rates significantly increased (*P* < 0.001).

**Table 1:** Clinical characteristics of all aTAAD patients.

Variable	All (*n* = 1,295, 100%)	AKI (*n* = 645, 49.8%)	No AKI (*n* = 650, 50.2%)	*P*-value
Preoperative				
Age, year	53.6 ± 13.3	54.0 ± 13.0	53.2 ± 13.7	.312
Male	976 (75.4%)	525 (81.4%)	451 (69.4%)	.000
BMI, kg/m^2^	25.8 ± 4.1	26.6 ± 3.9	25.0 ± 3.9	.000
Smoking	330 (25.5%)	189 (29.3%)	141 (21.7%)	.002
Drinking	233 (18.0%)	127 (19.7%)	106 (16.3%)	.129
Hypertension	970 (74.9%)	533 (82.6%)	437 (67.2%)	.000
Previous cardiovascular disease	143 (11.0%)	65 (10.1%)	78 (12%)	.288
CKD	45 (3.5%)	32 (5%)	13 (2%)	.004
Time from symptom onset to diagnosis, h	10 (7, 18)	9 (6, 15)	10 (7, 22)	.005
Hyperacute, <48 h	1,152 (89.0%)	594 (92.1%)	558 (85.8%)	.000
Preoperative hypotension	95 (7.3%)	52 (8.1%)	43 (6.6%)	.339
Intestinal malperfusion	53 (4.1%)	39 (6.0%)	14 (2.2%)	.000
Renal artery involved	572 (44.2%)	299 (46.4%)	273 (42%)	.0446
Renal hypoperfusion	146 (11.2%)	99 (15.3%)	47 (7.2%)	.000
Intraoperative				
Surgery time, min	450.4 ± 108.3	480.0 ± 114.1	426.3 ± 96.2	.000
CPB time, min	223.4 ± 63.9	235.3 ± 67.5	211.6 ± 57.8	.000
Cross-clamp time, min	159.6 ± 51.4	165.4 ± 53.7	153.9 ± 48.5	.000
Circulation arrest, min	29.3 ± 11.0	29.9 ± 11.2	28.8 ± 10.7	.085
Lowest temperature, ° C	21.4 ± 2.4	21.1 ± 2.4	21.6 ± 2.4	.000
Cannulation strategies				.268
Right axillary artery	202 (15.6%)	108 (16.7%)	94 (14.5%)	
Femoral artery	316 (24.4%)	144 (22.3%)	172 (26.5%)	
Femoral + axillary	719 (55.5%)	366 (56.7%)	353 (54.3%)	
Other	58 (4.5%)	27 (4.2%)	31 (4.8%)	
Stent implantation	1,062 (82.0%)	563 (87.3%)	499 (76.8%)	.000
Intraoperative urine output, ml/(h·kg)	2.4 (1.4, 3.7)	1.9 (1.2, 3.0)	2.8 (1.8, 4.3)	.000
Postoperative				
Myoglobin > 10 ULN	58 (4.5%)	55 (8.5%)	3 (0.5%)	.000
Requiring dialysis	186 (14.4%)	177 (27.4%)	9 (1.4%)	.000
Length of intubation, h	28 (13.3, 72)	52.5 (17.5, 115)	17 (11, 45.3)	.000
Delayed extubation	502 (38.8%)	342 (53.0%)	160 (24.6%)	.000
ICU stay, day	5 (3, 8)	6 (4, 11)	4 (2, 6)	.000
Hospital stay, day	18 (14, 24)	20 (15, 27)	17 (13, 21)	.000
30-day mortality	91 (7.0%)	77 (11.9%)	14 (2.2%)	.000

AKI, acute kidney injury; BMI, body mass index; CKD, chronic kidney disease; eGFR, estimated glomerular filtration rate; CPB, cardiopulmonary bypass; ULN, upper limit of normal value; ICU, intensive care unit.

* *P* < 0.05.

** *P* < 0.01.

*** *P* < 0.001.

### PO-AKI recovery patterns by eGFR trajectories

**Figure 2:**
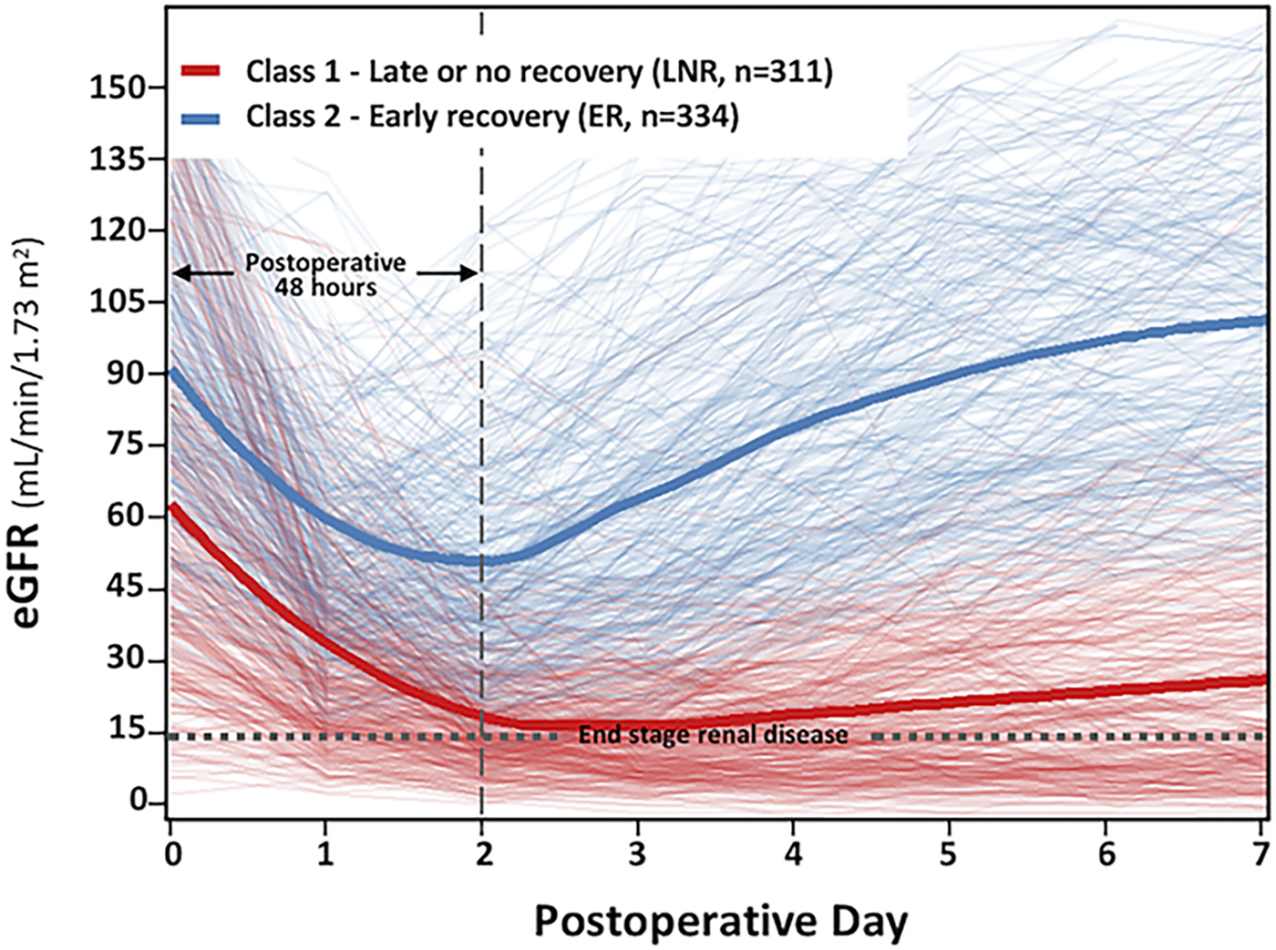
Class-specific and integrated estimated glomerular filtration rate (eGFR) trajectories stratified by class in the postoperative acute kidney injury (PO-AKI) cohort. This figure represents the main profiles of kidney function identified with latent class mixed models. Each patient, represented by an individual eGFR trajectory, is assigned to the class for which the membership probability is the highest. The cluster of *light red* lines in the background represents the individual patient eGFR trajectory with the highest probability assigned to Class 1 (late or no recovery, LNR, *n* = 311), and the cluster of *light blue* lines in the background represents the individual patient eGFR trajectory with the highest probability assigned to Class 2 [early recovery (ER) *n* = 334]. The *bold red* and *bold blue* lines represent the estimated mean eGFR trajectories in each class.

A total of 645 (49.8%) patients developed PO-AKI after open aTAAD surgery (Table 2). The primary diagnosis of AKI was reached within the first 48 h in 610 patients (94.6%), within the period of POD3 (the 3rd day post operation) in 25 patients (3.9%), within POD4-7 in the remaining 10 patients (1.6%). We used the unsupervised LCMM approach to depict and cluster the curvilinear eGFR trajectory of the 645 PO-AKI patients, and the best fit to data was obtained with two latent classes (Supplementary Table S2: 4-quant-spline, Model 2) corresponding to median eGFR trajectory 1 and trajectory 2 (Figure 2), with a mean posterior probability reaching 0.90 in Class 1 and 0.82 in Class 2, suggesting an overall good discrimination ability. Both trajectories had a sharp decrease (ΔeGFR_class1_ = −36.30 ml/min/1.73m^2^, ΔeGFR_class2_ = -41.30 ml/min/1.73m^2^, *P* = 0.39, Supplementary Tables S3,S4) within the first postoperative 48 h, which indicated that almost all patients have experienced severe kidney injury during the procedures, but the basic renal function and outcomes of the two classes are significantly different. Before surgery, the baseline median eGFR of Class 1 was significantly lower than Class 2 (56.40 vs. 88.75 ml/min/1.73m^2^, *p* < 0.001), which indicated a more severe injury of renal function in Class 1. After surgery, the eGFR of patients in Class 1 significantly declined followed by slow or no improvement. This was thus described as the late or no-recovery group (Class 1, LNR-AKI group, *n* = 311, 48.2%). Among them, only 149 patients (149/311, 47.9%) recovered baseline renal function at discharge, and 72 patients (72/311, 23.2%) remained to be dialysis-dependent. Instead, the eGFR of patients in Class 2 experienced a significant decline followed by a rapid rebound, which is thus described as the early-recovery group (Class 2, ER-AKI group, *n* = 334, 51.8%). Among them, 280 patients (83.8%) recovered baseline renal function at discharge, and no patients remained to be dialysis-dependent.

**Table 2:** Clinical characteristics by AKI recovery patterns.

Variable	LNR-AKI (*n* = 311, 48.2%)	ER-AKI (*n* = 334, 51.8%)	*P*-value
Preoperative			
Age, year	54.0 ± 13.3	54.0 ± 12.7	.988
Male	250 (80.4%)	275 (82.3%)	.545
BMI, kg/m^2^	26.8 ± 4.4	26.6 ± 4.0	.253
Smoking	94 (30.2%)	95 (28.4%)	.665
Drinking	62 (19.9%)	65 (19.5%)	.921
Hypertension	257 (82.6%)	276 (82.6%)	1.0
Previous cardiovascular disease	37 (11.9%)	28 (8.4%)	.151
CKD	22 (7.1%)	10 (3.0%)	.019
Time from symptom onset to diagnosis, h	9 (6, 13)	9.5 (6, 16.5)	.467
Hyperacute, <48 h	292 (93.9%)	302 (90.4%)	.110
Preoperative hypotension	27 (8.7%)	25 (7.5%)	.665
Intestinal malperfusion	23 (7.4%)	16 (4.8%)	.188
Renal artery involved	150 (48.2%)	149 (44.6%)	.052
Renal hypoperfusion	62 (19.9%)	37 (11.1%)	.006
Intraoperative			
Surgery time, min	498.1 ± 114.2	462.3 ± 108.5	.000
CPB time, min	246.3 ± 71.1	225.1 ± 62.3	.000
Cross-clamp time, min	171.7 ± 53.2	159.5 ± 53.5	.004
Circulation arrest, min	31.9 ± 11.8	28.0 ± 10.2	.000
Lowest temperature, °C	21.2 ± 2.5	21.1 ± 2.4	.802
Cannulation strategies			.885
Right axillary artery	49 (15.8%)	59 (17.7%)	
Femoral artery	70 (22.5%)	74 (22.2%)	
Femoral + axillary	180 (57.9%)	186 (55.7%)	
Other	12 (3.9%)	15 (4.5%)	
Stent implantation	274 (88.1%)	289 (86.5%)	.557
Intraoperative urine output, ml/(h·kg)	1.5 (0.8, 2.4)	2.4 (1.5, 3.4)	.000
Postoperative			
Myoglobin > 10 ULN	47 (15.1%)	8 (2.4%)	.000
Requiring dialysis	160 (51.4%)	17 (5.1%)	.000
Length of intubation, h	77 (27, 154)	37 (15.5, 72)	.000
Delayed extubation	205 (65.9%)	137 (47.0%)	.000
ICU stay, day	8 (5, 17)	5 (3, 8)	.000
Hospital stay, day	22 (16, 33)	18 (14, 24)	.000
30-day mortality	60 (19.3%)	17 (5.1%)	.000

AKI, acute kidney injury; LNR-AKI, late or no recovery of AKI; ER-AKI, early recovery of AKI; BMI, body mass index; CKD, chronic kidney disease; eGFR, estimated glomerular filtration rate; CPB, cardiopulmonary bypass; ULN, upper limit of normal value; ICU, intensive care unit.

* *P* < 0.05.

** *P* < 0.01.

*** *P* < 0.001.

### Association of clinical variables and short-term outcomes with eGFR trajectories

Of the 645 PO-AKI patients, 11.9% (71) died during hospitalization or a period of 30 days. LNR-AKI was significantly associated with an increased risk of all-cause death (19.3% vs. 5.1%, *P* < 0.001). Compared with ER-AKI, the odds ratio (OR) for mortality was 4.457 (95% CI: 2.538–7.830). Moreover, LNR-AKI was associated with more frequent adverse events (Table 2): including more use of CRRT (51.4% vs. 5.1%, *P* < 0.001), prolonged duration of mechanical ventilation (median: 77 vs. 37 h, *P* < 0.001), increased proportion of delayed extubation (65.9% vs. 47.0%, *P* < 0.001), longer ICU stay (median: 8 vs. 5 days, *P* < 0.001), and longer hospital stay (median: 22 vs. 18 days, *P* < 0.001).

We initially analyzed 32 clinical and functional factors for predicting trajectory subgroups at the first 24 h post operation (Table 2). Multicollinearity was assessed by calculating variance inflation factors (VIF). The variables with VIF > 10 were removed from the model. LNR-AKI was more likely than ER-AKI to have CKD history; renal hypoperfusion; longer duration of intraoperative procedures including surgery time, CPB time, cross-clamp time, and circulation arrest time; less intraoperative urine output; and higher level of postoperative myoglobin. Using backward stepwise variable selection, five variables significantly associated with subgroups were selected (Table 3), namely, CKD, renal hypoperfusion, circulation arrest time, intraoperative urine, and myoglobin > 10 ULN. Patients with preoperative CKD and CT indication of renal hypoperfusion had a 2.94-fold (OR = 2.94, 95% CI: 1.35–6.84) and 1.83-fold (OR = 1.83, 95% CI: 1.14–2.97) increase in the risk of poor renal recovery. The postoperative myoglobin (>10 ULN) was the strongest predictor, with an OR value reaching 5.775 when the LNR-AKI group was compared with the ER-AKI group (95% CI: 2.762–13.647, *P* < 0.001). Considering intraoperative factors, the risk of poor recovery of renal function increased by 2.8% for every minute of circulatory arrest time (OR = 1.028, 95% CI: 1.013–1.045, *P* < 0.001). The intraoperative urine production correlated inversely with the prognosis: for every 1 ml/kg/h increase in urine output, the risk decreased by 27.6% (OR = 0.724, 95% CI: 0.641–0.812, *P* < 0.001).

**Table 3:** Determinants and associations with AKI recovery patterns.

Variable	*β*	SE	*P*	OR^a^	95% CI	
					Lower	Upper
Preoperative						
CKD	1.078	0.410	0.009	2.940	1.349	6.839
Renal hypoperfusion	0.604	0.244	0.013	1.830	1.140	2.970
Intraoperative						
Circulation arrest time	0.028	0.008	<0.001	1.028	1.013	1.045
Intraoperative urine	−0.323	0.060	<0.001	0.724	0.641	0.812
Postoperative						
Myoglobin > 10ULN	1.753	0.403	<0.001	5.775	2.762	13.647

AKI, acute kidney injury; CKD, chronic kidney disease; ULN, upper limit of normal value.

^a^ Early-recovery AKI group was the reference.

### Establishment and assessment of the prediction model

**Figure 3:**
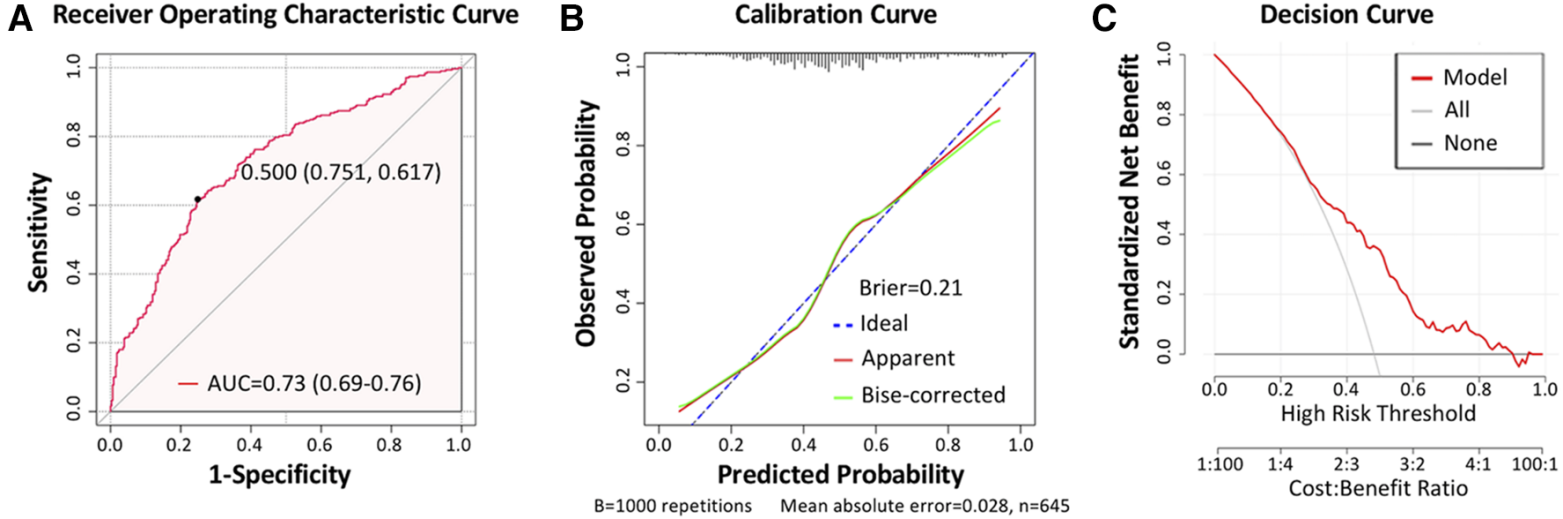
Model assessment. (**A**) Receiver operating characteristic (ROC) curve of the prediction model, with the area under curve (AUC) reaching 0.73 (95% CI: 0.69–0.76), sensitivity of 61.74%, and specificity of 75.15%. (**B**) Calibration curve of the prediction model, with the Brier score being 0.21. (**C**) Decision curve of the prediction model.

**Figure 4:**
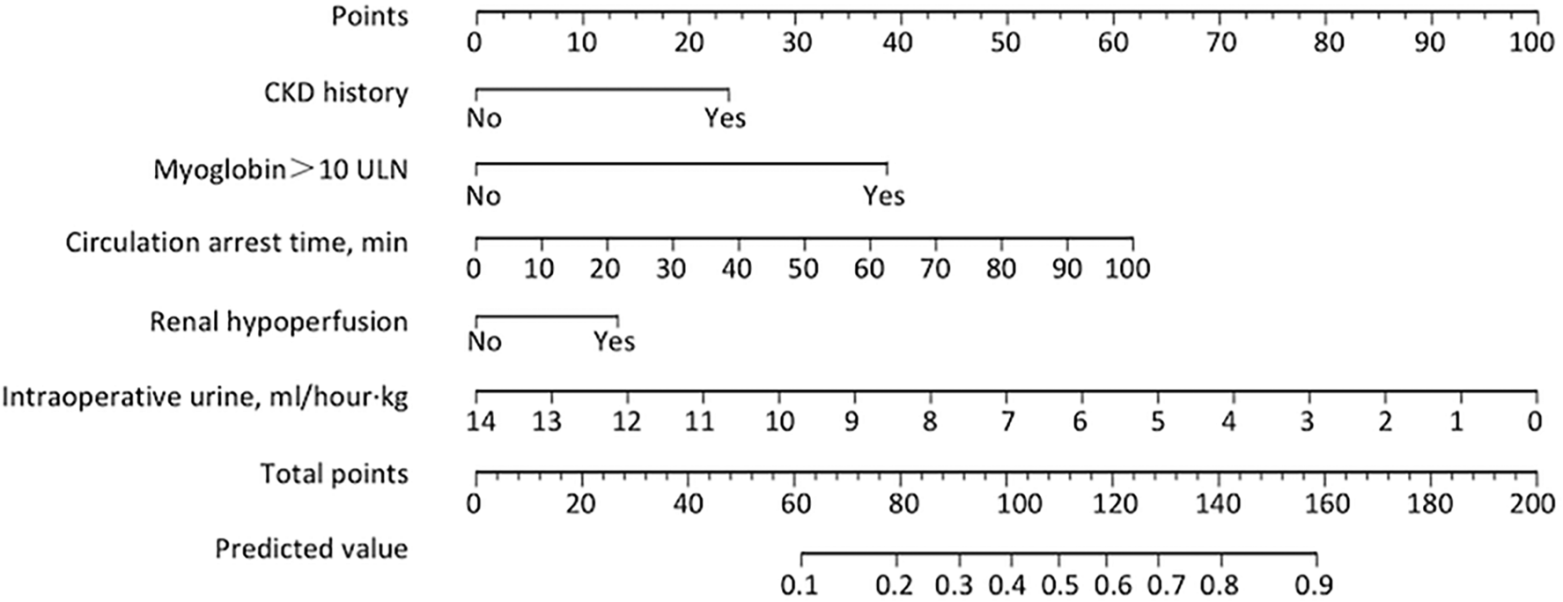
Nomogram for predicting risks of progression into LNR-AKI. To use the nomogram for an individual patient, first draw a vertical line to the top points row to assign points for each variable; then, add the points from each variable together and drop a vertical line from the total points row to obtain the predicted risk value of progression into LNR-AKI. LNR-AKI, late or no recovery of acute kidney injury.

Next, we established a separate model for predicting the recovery trajectory of the PO-AKI with the regression coefficient data from the multivariable regression analyses. The AUC of the prediction model was 0.73 (95% CI: 0.69–0.76, Figure 3A), and the sensitivity and specificity were 61.74% and 75.15%, respectively. The calibration curve indicated that the predicted incidence was consistent with the observed incidence of subgroups (Figure 3B), and the Brier score was 0.21. For internal validation of the PO-AKI cohort, we used bootstrap sampling 1,000 times and derived average values of AUC of 0.73 and Brier score of 0.21. Decision curve analysis showed that our model can add net benefits for patients (Figure 3C). For the convenience of practical use of the predictive model, we constructed the nomogram for clinicians (Figure 4).

## Discussion

In this large retrospective study of patients with aTAAD, we highlight a novel way to map for the first time non-linear eGFR trajectory after the occurrence of PO-AKI and classify individuals into distinct, mutually exclusive groups in an unsupervised approach. Hence, this strategy not only helps to conceptualize the short-term trajectories of eGFR but also allows us to analyze the population heterogeneity and its determinants, to identify persons with a high risk of rapid progression for early prevention and intensive treatment.

Our data suggest that up to 50% of the patients could experience varying degrees of renal injury after aTAAD surgery, which is consistent with recorded prevalence (4). The kidney is an organ susceptible to ischemia-reperfusion injury (12). The decrease in renal perfusion or even renal infarction caused by aortic dissection is the first blow to renal function, while the second blow is the non-pulsatile low perfusion blood during deep hypothermic circulatory arrest surgery. Therefore, the kidney of patients with aortic dissection often experiences several hours of ischemia and subsequent reperfusion injury, making postoperative AKI extremely common.

LNR-AKI was significantly associated with worse clinical outcomes. Previous studies commonly used Risk, Injury, Failure, Loss, and End-stage kidney disease (RIFLE) (13) or Acute Kidney Injury Network (14) criteria to define the stage of AKI and found that the severity of AKI was significantly correlated with risk of mortality. Our results indicated lower baseline eGFR in LNR-AKI, while lower baseline eGFR and poor recovery of renal function at discharge were both associated with the long-term incidence of CKD stage (15, 16). Previous studies have shown that the maximum SCr concentration within 72 h after AKI diagnosis was not significantly different between the resolving and non-resolving AKI groups (17). Similarly in our cohort, patients in both groups had a rapid eGFR decline of nearly 40 ml/min/1.73 m^2^ within postoperative 48 h, but the recovery pattern of eGFR was significantly heterogeneous, which cannot be discriminated by traditional longitudinal and linear models. This study employed an unsupervised model LCMM to identify different subgroups in relation to different clinical disease progressions, by which further heterogeneous characterization could provide a more prognosis-relevant stratification. In addition, it would be of particular interest to further use the LCMM analysis in long-term follow-up data to predict the individuals with risks for “CKD on AKI.”

This study employed repeated eGFR measurements, instead of SCr, as a key biomarker used in the LCMM algorithm for better performance in the mathematical model. LCMM is an unsupervised clustering analysis, and its clustering effect depends on the differences between samples. Patients with PO-AKI, especially those undergoing CRRT treatment, have creatinine variation similar to a sine curve, with a larger range of variation than eGFR (Supplementary Tables S3,S4), and this fluctuation is not a true reflection of renal function. Most importantly, this study does not focus on the precise evaluation of renal function at a certain point in time but rather attempts to simulate the development curve of renal function. Compared with SCr, eGFR performs more stably in LCMM. On the other hand, we acknowledge that the traditional renal function indicators commonly used in clinical practice, such as SCr, urea nitrogen (BUN), and eGFR, are universally neither sensitive nor accurate indicators for evaluating renal function injury at an early stage. The level of SCr is influenced by age, gender, muscle content, dietary intake, etc., and the concentration of SCr begins to increase when GFR decreases by ∼50%. Meanwhile, for patients with severe CKD, the accuracy of SCr is also affected by factors such as renal tubular secretion and gastrointestinal degradation. At present, there are some new biomarkers, such as cystatin C (CysC), neutrophil gelatinase-associated lipocalin (NGAL), and kidney injury molecule 1 (KIM-1), which are the most sensitive indicators for early diagnosis of acute renal failure and have higher specificity for ischemic or nephrotoxic kidney disease, but most of them have not been widely used in clinical practice. In future research, we will add those multidimensional indicators to help evaluate renal function more comprehensively and accurately.

This study identified several critical determinants for risk stratifying patients with PO-AKI. Firstly, organ malperfusion manifested as a severe clinical complication, and the number of malperfused organs is positively correlated with 30-day mortality (12). Our data confirmed that preoperative renal malperfusion (CT hypoenhancement sign in renal cortex) was an important determinant for renal poor recovery. Hemodynamic changes caused by aortic dissection can lead to a redistribution of blood flow throughout the body, including the kidneys. Previous studies have illustrated that the blood flow to the kidneys is directly related to the type of aortic dissection, renal artery origins, the number and size of the intimal tears, and false lumen thrombosis (18) and is commonly lower than the values of normal kidneys using the same CT system (19, 20). Meanwhile, CT findings of renal artery dissection and renal hypoenhancement signs could predict long-term renal atrophy, which howbeit could be mitigated by surgical treatment such as endovascular aneurysm repair (EVAR) (11). Secondly, the significantly increased level of myoglobin (indication of limb malperfusion) presented as the strongest predictor for LNR-AKI. Not surprisingly, ischemic necrosis of muscles, caused by limb branch artery dissection or decreased aortic perfusion blood flow, can promote the release of creatine kinase (CK), myoglobin, free calcium, etc. Myoglobin is the principal compound corresponding to renal failure, which induces renal tubular obstruction, tubular necrosis, and lipid peroxidation, especially in the presence of insufficient blood volume and acidic urine (21–25). However, the extensive myocardial injury and general elevation of CK after cardiac surgery leads to a decrease in the diagnostic specificity of CK for limb ischemia. For more data missing of CK in our retrospective cohort, we employed the variable “myoglobin” for predicting renal recovery, and the results prompted that myoglobin may be a very ideal indicator for predicting limb ischemia-related renal failure in aTAAD surgery.

Our results showed that the increased circulation arrest was correlated with worse renal recovery (Table 3). Numerous studies have demonstrated that CPB of cardiac surgery exacerbates the burden of PO-AKI by a complex and multifactorial etiology, including decreased renal perfusion pressure, decreased renal blood flow, deficient oxygenation delivery, and activation of multiple pro-inflammatory pathways (26–29). Intraoperative CPB time, cross-clamp time, and circulatory arrest time are all critical determinants. Due to the collinear relationship between these three variables, we chose the “circulatory arrest time” with the highest correlation coefficient for the best performance of the prediction model.

In combination with SCr, urine output remains the cornerstone for diagnosing AKI, which prompts us of the great importance of monitoring intraoperative urine for predicting the progression of PO-AKI. Hence, we included the parameter of intraoperative hourly urine per kilogram body weight in the analysis, and the result proved excellent performance in discriminating different recovery groups (Table 3). The perfusion pressure of renal parenchymal capillary bed is determined by the difference of arterial pressure minus venous pressure, so any factors that promote reduction in arterial pressure, or increase in renal venous pressure (such as volume overload, severe heart failure, or abdominal compartment syndrome) may cause renal hypoperfusion and increase the AKI severity (30–32).

The limitations of this study should be considered. Firstly, AD patients were transferred from vast peripheral areas by emergency, almost all of whom lacked eGFR data within the past three months for evaluating the baseline renal function, but the hemodynamic instability in AD often reduces renal perfusion; therefore, the “baseline eGFR” measured upon hospitalization did not represent the real state of basis renal function before the onset. Patients who died within postoperative 48 h were excluded for unavailable AKI diagnosis and recovery assessment, but exclusion of these early deceased patients, who often suffer from multiple organ failure including severe renal insufficiency, could increase selection bias and lead to an overestimation of median eGFR. Secondly, statistical performance is not excellent in this study partly due to the inherent limitations of retrospective cohort studies and we expected more effective predictors, we therein suggested that clinicians or academics should consider carefully when drawing on the model and should actively focus on new key risk factors, which are important for further improving the diagnostic efficiency of predictive models.

In conclusion, this large cohort retrospective study employed an unsupervised approach to analyze and characterize eGFR trajectories of PO-AKI in aTAAD populations and defined two recovery patterns of PO-AKI with marked heterogeneity regarding risk for ICU-specific adverse events and 30-day mortality. In the future, trajectory-based AKI recovery phenotypes among aTAAD patients may allow for improved risk stratification and facilitate intensive monitoring and individualized treatment.

## Data Availability

The original contributions presented in the study are included in the article/supplementary materials, further inquiries can be directed to the corresponding author.
