# Supplementary material for: Development and validation of LCMM prediction algorithms to estimate recovery pattern of postoperative AKI in type A aortic dissection: a retrospective study
Source: Front Cardiovasc Med. 2024 Apr 19;11:1364332. doi: 10.3389/fcvm.2024.1364332 (PMC11066321; doi:10.3389/fcvm.2024.1364332)

**Supplementary Materials**

**Supplementary Table 1.** Variables missing information in the analysis sample

| **Variables** | **Missing number** | **Missing proportion (%)** |
| --- | --- | --- |
| Age | 2 | 0.3 |
| BMI | 18 | 2.8 |
| Smoking | 10 | 1.6 |
| Drinking | 3 | 0.5 |
| CKD | 6 | 0.9 |
| Time from symptom onset to diagnosis | 29 | 4.5 |
| Myoglobin > 10ULN | 53 | 8.2 |
| With femoral cannulation | 7 | 1.1 |

**Supplementary Table 2.** Fit statistics of latent class mixture model for eGFR

| Trajectory shape | Number of classes estimated | AIC | BIC | Class membership (%) | Mean posterior probabilities |
| --- | --- | --- | --- | --- | --- |
| Linear | 1 | 46618.60 | 46645.41 | Class 1 = 100.00 | Class 1 = 1.00 |
|  | 2 | 46480.17 | 46524.86 | Class 1 = 24.65 Class 2 = 75.35 | Class 1 = 0.80 Class 2 = 0.97 |
|  | 3 | 46437.25 | 46499.82 | Class 1 = 22.63 Class 2 = 24.81 Class 3 = 52.56 | Class 1 = 0.82 Class 2 = 0.89 Class 3 =0.88 |
|  | 4 | 46380.38 | 46460.83 | Class 1 = 38.91 Class 2 = 25.27 Class 3 = 24.96 Class 4 = 10.85 | Class 1 = 0.90 Class 2 = 0.73 Class 3 = 0.86 Class 4 = 0.81 |
|  | 5 | 46379.07 | 46477.39 | Class 1 = 24.81 Class 2 = 6.51 Class 3 = 32.40 Class 4 = 25.89 Class 5 = 10.39 | Class 1 = 0.86 Class 2 = 0.54 Class 3 = 0.77 Class 4 = 0.77 Class 5 = 0.82 |
| 3-quant-spline | 1 | 44255.30 | 44295.52 | Class 1 = 100.00 | Class 1 = 1.00 |
|  | 2 | 44163.14 | 44221.24 | Class 1 = 47.13 Class 2 = 52.87 | Class 1 = 0.91 Class 2 = 0.83 |
|  | 3 | 44133.56 | 44209.54 | Class 1 = 6.98 Class 2 = 49.92 Class 3 = 43.10 | Class 1 = 0.80 Class 2 = 0.78 Class 3 = 0.83 |
|  | 4 | 44126.74 | 44220.60 | Class 1 = 5.12 Class 2 = 53.33 Class 3 = 25.43 Class 4 = 16.12 | Class 1 = 0.84 Class 2 = 0.79 Class 3 = 0.79 Class 4 = 0.74 |
|  | 5 | 44116.08 | 44227.82 | Class 1 = 4.65 Class 2 = 9.46 Class 3 = 61.40 Class 4 = 14.57 Class 5 = 9.92 | Class 1 = 0.87 Class 2 = 0.78 Class 3 = 0.89 Class 4 = 0.75 Class 5 = 0.79 |
| 4-quant-spline | 1 | 44114.77 | 44159.46 | Class 1 = 100.00 | Class 1 = 1.00 |
|  | 2 | 44029.64 | 44092.21 | Class 1 = 48.22 Class 2 = 51.78 | Class 1 = 0.90 Class 2 = 0.82 |
|  | 3 | 44003.29 | 44083.74 | Class 1 = 5.58 Class 2 = 48.22 Class 3 = 46.20 | Class 1 = 0.83 Class 2 = 0.77 Class 3 = 0.84 |
|  | 4 | 43996.29 | 44094.61 | Class 1 = 4.96 Class 2 = 15.82 Class 3 = 53.64 Class 4 = 25.58 | Class 1 = 0.85 Class 2 = 0.72 Class 3 = 0.78 Class 4 = 0.80 |
|  | 5 | 43991.76 | 44107.96 | Class 1 = 4.96 Class 2 = 23.10 Class 3 = 26.98 Class 4 = 27.92 Class 5 = 17.05 | Class 1 = 0.85 Class 2 = 0.76 Class 3 = 0.63 Class 4 = 0.80 Class 5 = 0.71 |

**Supplementary Table 3**. Distribution of eGFRs.

| **eGFR** | **Class 1** | | | |  | **Class2** | | | |
| --- | --- | --- | --- | --- | --- | --- | --- | --- | --- |
|  | **25%** | **50%** | **75%** | **IQR** |  | **25%** | **50%** | **75%** | **IQR** |
| D0 | 35.70 | 56.40 | 85.10 | 49.40 |  | 69.35 | 88.75 | 110.10 | 40.75 |
| D1 | 20.70 | 29.30 | 42.40 | 21.70 |  | 40.50 | 50.95 | 64.80 | 24.30 |
| D2 | 15.80 | 20.10 | 27.60 | 11.80 |  | 35.98 | 47.45 | 65.15 | 28.18 |
| D3 | 13.80 | 19.50 | 27.40 | 13.60 |  | 43.90 | 59.95 | 79.60 | 35.70 |
| D4 | 14.00 | 21.05 | 31.05 | 17.05 |  | 55.00 | 74.45 | 95.75 | 40.75 |
| D5 | 14.10 | 22.30 | 35.90 | 21.80 |  | 65.75 | 84.35 | 108.60 | 42.85 |
| D6 | 14.00 | 24.50 | 39.40 | 25.40 |  | 70.00 | 92.30 | 115.00 | 45.00 |
| D7 | 15.00 | 25.80 | 41.35 | 26.35 |  | 74.78 | 96.70 | 120.00 | 45.22 |

**Supplementary Table 4**. Distribution of SCr.

| **SCr** | **Class 1** | | | |  | **Class2** | | | |
| --- | --- | --- | --- | --- | --- | --- | --- | --- | --- |
|  | **25%** | **50%** | **75%** | **IQR** |  | **25%** | **50%** | **75%** | **IQR** |
| D0 | 72.03 | 100.35 | 150.75 | 78.72 |  | 63.85 | 80.00 | 100.30 | 36.45 |
| D1 | 119.00 | 165.00 | 235.00 | 116.00 |  | 102.00 | 122.00 | 159.00 | 57.00 |
| D2 | 164.00 | 241.00 | 329.50 | 165.50 |  | 108.00 | 148.00 | 196.00 | 88.00 |
| D3 | 168.25 | 246.00 | 359.75 | 191.50 |  | 90.00 | 121.00 | 175.00 | 85.00 |
| D4 | 152.00 | 227.00 | 329.25 | 177.25 |  | 72.25 | 96.50 | 132.00 | 59.75 |
| D5 | 138.50 | 195.00 | 303.00 | 164.50 |  | 67.50 | 86.50 | 113.00 | 45.50 |
| D6 | 126.00 | 174.00 | 275.00 | 149.00 |  | 64.00 | 78.00 | 102.00 | 38.00 |
| D7 | 121.50 | 181.00 | 281.50 | 160.00 |  | 65.00 | 80.00 | 108.00 | 43.00 |

**Supplementary Figure 1.** Distribution of missing variables (A), and results after imputation (B).


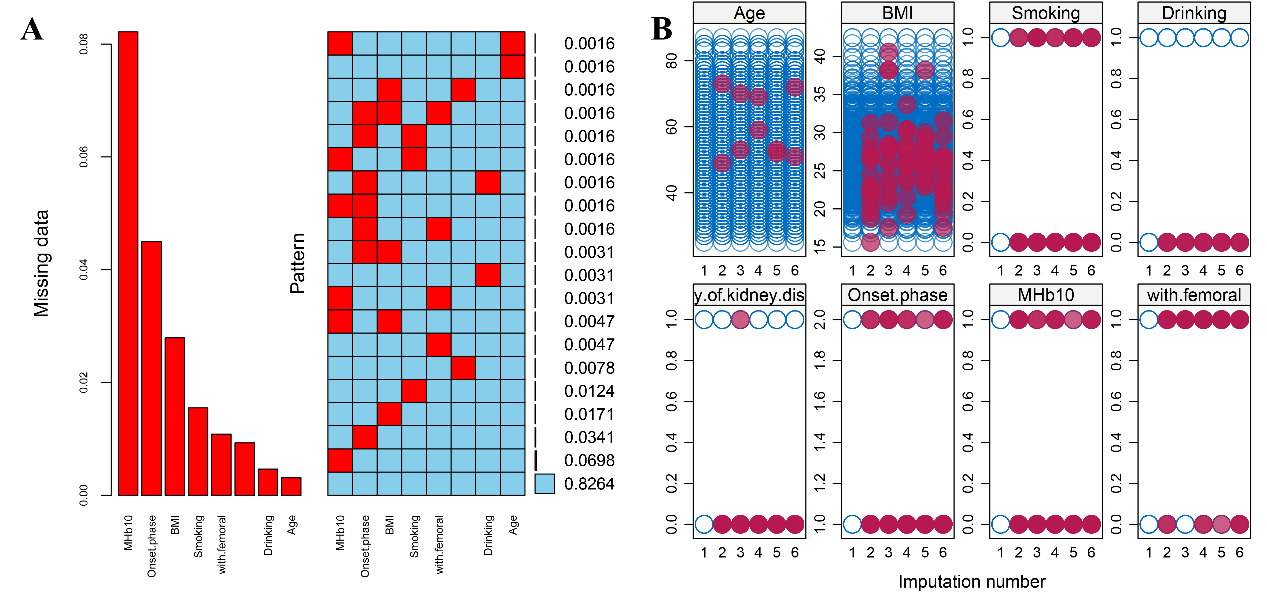

Supplement: Supplementary file 1 [file Datasheet1.docx]
